# Pharmacogenetics of Carbamazepine and Valproate: Focus on Polymorphisms of Drug Metabolizing Enzymes and Transporters

**DOI:** 10.3390/ph14030204

**Published:** 2021-03-01

**Authors:** Teresa Iannaccone, Carmine Sellitto, Valentina Manzo, Francesca Colucci, Valentina Giudice, Berenice Stefanelli, Antonio Iuliano, Giulio Corrivetti, Amelia Filippelli

**Affiliations:** 1 Department of Medicine, Surgery and Dentistry “Scuola Medica Salernitana”, University of Salerno, 84081 Baronissi, Italy; tiannaccone@unisa.it (T.I.); vmanzo@unisa.it (V.M.); francescacolucci90@gmail.com (F.C.); vgiudice@unisa.it (V.G.); b.stefanelli@studenti.unisa.it (B.S.); a.iuliano@hotmail.it (A.I.); afilippelli@unisa.it (A.F.); 2 Clinical Pharmacology and Pharmacogenetics Unit, University Hospital “San Giovanni di Dio e Ruggi d’Aragona”, 84131 Salerno, Italy; 3 European Biomedical Research Institute of Salerno (EBRIS), 84125 Salerno, Italy; corrivetti@gmail.com

**Keywords:** pharmacogenomics, carbamazepine, valproate, mood stabilizers

## Abstract

Pharmacogenomics can identify polymorphisms in genes involved in drug pharmacokinetics and pharmacodynamics determining differences in efficacy and safety and causing inter-individual variability in drug response. Therefore, pharmacogenomics can help clinicians in optimizing therapy based on patient’s genotype, also in psychiatric and neurological settings. However, pharmacogenetic screenings for psychotropic drugs are not routinely employed in diagnosis and monitoring of patients treated with mood stabilizers, such as carbamazepine and valproate, because their benefit in clinical practice is still controversial. In this review, we summarize the current knowledge on pharmacogenetic biomarkers of these anticonvulsant drugs.

## 1. Introduction

Mood stabilizers are a heterogeneous class of drugs often prescribed in association for treatment of bipolar disorder (BD) which affects 5–6% of general population and represents one of the top 20 causes of disability worldwide [1,2]. BD, a mental health condition, is characterized by unusual, excessive, and unforeseeable mood swings that can dramatically worsen the quality of life of affected patients. The main clinical manifestations of BD are episodes of depression alternated with periods of abnormally elevated mood without or with psychotic symptoms, also named mania. Productive mania can be successfully treated with lithium, while other disorders, like acute bipolar depression and rapid cycling disease, can be more efficiently treated with mood stabilizers, such as two anticonvulsant drugs, carbamazepine (CBZ) and valproate (VPA). These two drugs are also effective in prophylaxis of recurrent BD in children and adolescents [3,4].

Response to CBZ and VPA for BD treatment is highly variable because of clinical and biological heterogeneity of diseases, pharmacokinetics/pharmacodynamics (PK/PD) modifications related to polytherapy, the presence of comorbidities, and poor adherence to treatment [5,6]. Enzymes involved in drug metabolism can have different efficacy and kinetics in drug transformation. These variabilities can be related to the presence of polymorphisms in related genes, mainly single nucleotide polymorphisms (SNPs), which can modify gene and consequently protein sequence making translated protein more or less efficient compared to the“wild-type.” Pharmacogenetics studies the influence of those SNPs on drug response in order to tailor therapy. For example, if a patient is a so-called “slow-metabolizer” for a drug because one of the key-enzyme in the metabolic way is less efficient, patient can accumulate either an active or inactive metabolite in body fluids. In both cases, patient can experience drug toxicity due to increased levels of active metabolite, or reduced clearance of inactive molecules. Therefore, by knowing in advance if a subject is a slow- or fast-metabolizer, the clinician can choose the appropriate drug dosage to ensure maximum clinical benefits while minimizing the side effects. Over the past decade, our knowledge of pharmacogenomics in psychotropic drugs has increased significantly. Several genetic polymorphisms including genes involved in PK/PD can be related to variability in CBZ and VPA efficacy, safety, and drug resistance which affects >30% of epileptic patients and >20% of BD subjects [7,8]. Pharmacogenetic biomarkers could help clinicians in personalizing neuropsychiatric therapy in those patients. This review provides an overview on current pharmacogenomics biomarkers of responsiveness to the two most anticonvulsants used as mood stabilizers, CBZ and VPA, in BD treatment.

## 2. CBZ and VPA Mechanisms of Action

BD, also known as manic-depressive illness or manic depression, is a psychiatric disorder including three different conditions, bipolar I, bipolar II, and cyclothymic disorder, based on the recurrence of mania/hypomania and/or major depressive episodes. Rapid-cycling BD is defined when four or more episodes occur within a 12-month period [9,10,11]. Goals of BD treatment consist in managing acute mania and long-term prevention of relapses. Until the discovery of lithium, electroconvulsive therapy had been the most effective treatment of mania also showing anticonvulsant effects by increasing seizure threshold and decreasing manic symptoms [12,13].

Based on the theory of kindling, the recurrence of manic states could expose patients to further manic episodes in BD as well as the appearance of repeated seizures could expose patients to further seizures in epilepsy. CBZ and VPA are gamma-aminobutyric acid (GABA)-mimetic drugs: CBZ is a positive allosteric modulator of GABA-A receptor, while VPA increases GABA release by blocking enzymes involved in GABA catabolism [14,15]. VPA is the most effective treatment for rapid cyclers’ patients. To explain its mood-stabilizing activity in mania, it has been hypothesized that VPA reduces the overstimulation of excitatory neurotransmission, by blocking the voltage-dependent sodium channels. It may alter the phosphorylation of ionic channels and modify their sensitivity; less sodium enters and there is a reduction in glutamate release. In addition, VPA enhances GABAergic tone, decreasing its reuptake, increasing its release, and slowing its degradation. These VPA synergic actions may explain its antimanic effect [16,17]. Like VPA, CBZ is used in chronic but not acute administration in BD and it is associated with the up-regulation of GABA-B receptors in the hippocampus, as a potential mechanism of mood stabilization. CBZ exerts anti-glutamatergic action decreasing glutamate’s postsynaptic efficacy by inhibiting intracellular calcium influx. Antimanic effect is linked to its glutamate antagonism [18]. A hypothesis regarding mood stabilizers action in BD is the “arachidonic acid” (AA) cascade theory. Like lithium, VPA and CBZ decreased AA turnover in the rat brain. These drugs could reduce manic symptoms by down-regulating brain AA metabolism because mania arises from excessive dopaminergic or glutamatergic, and reduced cholinergic signaling. This latter neurotransmission uses AA as a second messenger [19,20]. CBZ and lithium block D2-like receptor-initiated AA pathway. Their chronic use reduces D2 receptors density and D2-like receptor phosphorylation. Dopamine levels have been reported to be increased in the prefrontal cortex in BD patients [21]. VPA, CBZ and lithium block NMDA (N-methyl-D-aspartate) receptor-initiated AA pathway. NMDA receptors expression, distribution, and function are atypical in mood disorders. Inhibition of NMDA-induced Ca^2+^ influxes could explain the positive therapeutic effect of NMDA antagonism on bipolar depression [22]. In addition, intracellular aberration of Ca^2+^ signaling is related to BD episodes. Altered levels of calcium in cerebrospinal fluid were found in patients with mania and the long-term lithium treatment was associated with a dysregulation of calcium metabolism, such as hyperparathyroidism. CBZ and VPA could exert their effects also by directly modulating voltage-dependent calcium channels. Ca^2+^ channels are particularly represented on pre-synaptic neurons, are involved in neuronal depolarization and play an important role in epilepsy [23,24]. For example, in absence seizure, sensory cerebral cortex (SCX), ventrobasal posterior thalamic groups (VB), and reticular thalamic nucleus (RTN) are in an oscillatory loop in which RTN neurons are hyperpolarized and burst-fire through de-inactivation of T-type channels. Burst-firing of RTN neurons causes hyperpolarization of VB through GABA activation and T-type Ca^2+^ channel deactivation, inducing a rebound burst from the corticothalamic area and opening of de-inactivated Ca^2+^ channels. This rebound burst causes SCX depolarization and excitatory bursts toward the thalamus sustaining absence seizure. Therefore, the Ca^2+^ channel blockade is one of the therapeutic strategies for epilepsy treatment, and mood stabilizer with this property can be used as antiepileptic agents [25,26].

Several studies have described the efficacy and safety of extended-release CBZ in bipolar patients with acute manic or mixed episodes especially type I with response rates comparable to those obtained with lithium, VPA and atypical antipsychotics [27,28,29,30]. Moreover, CBZ and VPA can be effectively used for prophylaxis of BD episodes in lithium intolerance or nonresponsive patients, such as rapid cyclers or patients with mixed manic episodes and associated neuropsychiatric comorbidities [31,32]. However, CBZ as monotherapy shows less efficacy compared to lithium in treatment-naive bipolar patients with (hypo)manic episode, and displays less tolerability compared to VPA [33,34]. VPA is administered for treatment of adults and children with epilepsy, such as complex partial, simple, and absence seizures, and migraine headache prophylaxis. In addition, VPA might have histone deacetylase (HDAC) inhibitor properties and might be used for treatment of some cancers, in acquired immunodeficiency syndrome (AIDS), or neurodegenerative diseases [35]. VPA can be employed in managing mood episodes in patients who do not tolerate or are less responsive to lithium and is recommended as the first-line therapy in rapid cycling bipolar I disorder [36,37]. VPA is effective in mixed episodes and in depression prophylaxis showing similar efficacy compared to lithium and can be used in monotherapy or in combination with atypical antipsychotics [38,39,40]. However, treatment fails in 85% of the cases especially those who are treated with single agents. Annual frequency of recurrences can significantly decrease in rapid-cycling or cyclothymic patients using lithium and VPA or CBZ compared to those subjects treated with VPA or CBZ alone, probably because of synergistic effects on signaling pathways involved in neuroplasticity and neuroprotection [41,42]. Regarding safety profile, lithium plus CBZ is safer than lithium plus VPA showing less metabolic side effects: patients treated with CBZ show normal levels of high-density lipoprotein (HDL) cholesterol or blood glucose concentrations compared to patients resorting to VPA which had decreased HDL cholesterol and higher blood glucose. In addition, females on VPA treatment might have higher risk of developing metabolic syndrome compared to males [43,44].

## 3. Genetic Polymorphisms of Drug Metabolizing Enzymes and CBZ PK

**Figure 1:**
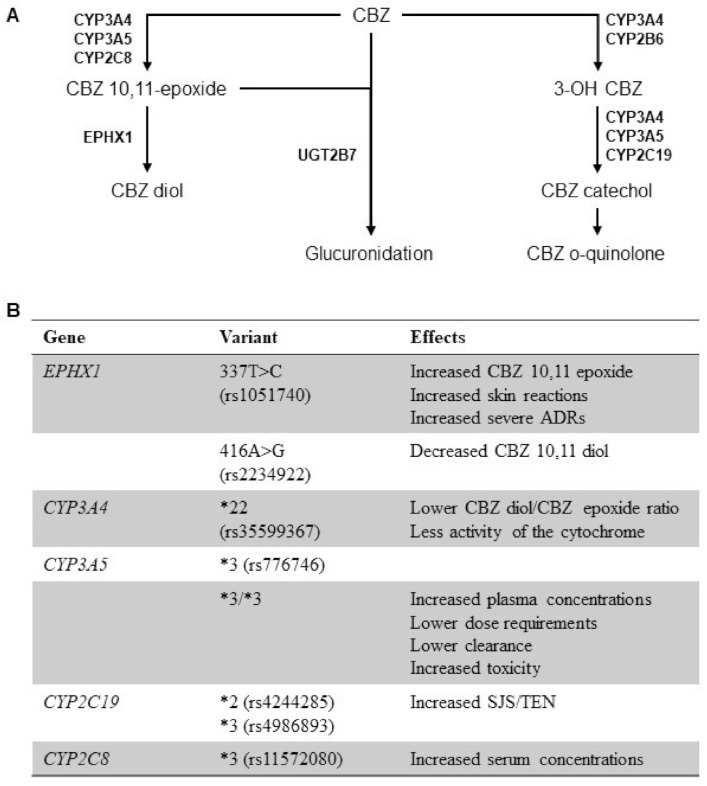
Pharmacokinetics (PK) and pharmacogenomics of carbamazepine (CBZ). (**A**) Liver metabolism of CBZ by cytochrome P450 (CYP) enzymes, epoxide hydrolase 1 (*EPXH1*), and UDP-glucuronosyltransferase (UGT) and relative metabolites. (**B**) Genetic polymorphisms in genes involved in CBZ metabolism and their effects on PK of the drug. Abbreviations. (*) allele nomenclature; ADR, adverse drug reactions; SJS/TEN, Steven-Johnson syndrome/toxic epidermal necrolysis.

Individual and ethnic variabilities influence the clinical efficacy and safety of CBZ partially explained by the presence of polymorphisms in genes encoding enzymes involved in phase I (cytochrome P450 (*CYP*)*3A4*, *CYP3A5, CYP2C8* and *EPHX1*) and phase II (*UGT2B7*) metabolism, transporters (*ABCB1* and *ABCC2*) or ion channels (Figure 1) [45,46]. However, further investigation is required to clarify the role of these polymorphisms as potential biomarkers of responsiveness to CBZ therapy. Among CYPs, *CYP3A4* and *CYP2C8* play an important role in CBZ metabolism and in CBZ 10–11 epoxide formation, the active equipotent CBZ metabolite. Another important CBZ metabolizer is the microsomal epoxide hydrolase encoded by the epoxide hydrolase 1 (*EPHX1)* gene. *EPHX1* is the major CBZ metabolizer responsible for the conversion of CBZ 10,11-epoxide in the inactive water-soluble metabolite CBZ 10,11-diol [47]. CBZ is also metabolized by glucuronidation mainly through the *UGT2B7* enzyme, or as catechol and o-quinolone by other CYPs, such as *CYP3A5* and *CYP2C19*. 

The *EPHX1* gene is polymorphic and is located on chromosome 1q42 with 9 exons and 8 introns. Two common polymorphic sites in the gene affect *EPHX1* activity and CBZ plasma levels. The tyrosine to histidine substitution in exon 3 (337T>C, rs1051740) could decrease the enzyme activity and the histidine to arginine substitution in exon 4 (416 A>G, rs2234922) could increase the enzyme activity. The CBZ Diol: CBZ Epoxide ratio is considered as a sensitive indicator of hydrolase catalytic activity. Moreover, the rs1051740 polymorphism has been associated with a higher Diol/Epoxide ratio and the rs2234922 polymorphism with a lower ratio [48]. The CC genotype of rs1051740 polymorphism was associated with an increase of CBZ 10,11-epoxide compared to the TT and CT+TT genotypes [49]. The GG genotype of rs2234922 was associated with a decreased serum concentration of CBZ-10,11-trans dihydrodiol and reduced CBZD:CBZE ratio, as reported in a metanalysis [50]. In addition, the C allele, and the C-G diplotype of 337T>C polymorphism might play an important role in increasing the risk of developing the adverse drug reactions (ADRs), such as Stevens-Johnson syndrome and toxic epidermal necrolysis, by increasing the concentration of a CBZ 10,11-epoxide in patients with epilepsy [51]. Like *EPHX1* polymorphisms, *CYP3A4* is involved in the regulation of metabolic enzyme activity. The CYP3A4*22 (rs35599367) polymorphism was significantly associated with lower CBZ diol/CBZ epoxide ratio and with less activity of the cytochrome, influencing inter-individual variability of CBZ metabolism [52]. However, there are conflicting reports in the literature regarding the influence of those polymorphisms in CBZ PK. Comparing serum CBZ 10,11-epoxide levels 4 h after drug administration, no statistically significant difference was found between the *EPHX1* 337 CC, CT, and TT genotypes. Similarly, no difference in serum CBZ 10,11-epoxide levels was observed between 416A>G *EPHX1* AA and AG genotypes; no difference also between CYP3A4*22 CC and CT genotypes was reported. The influence of *EPHX1* and *CYP3A4* polymorphisms on the metabolism of CBZ is still under investigation [53].

*CYP3A4* and *CYP3A5* show similar structure and substrate specificity. *CYP3A5* is highly polymorphic and the presence of CYP3A5*3 allele (6986A>G, rs776746), the most common nonfunctional variant, influences plasma CBZ levels [54]. Patients carrying CYP 3A5*3/*3 had significantly higher levels of CBZ plasma concentration compared to CYP3A5*1/*1 or CYP3A5*1/*3 carriers. Lower dose requirements and lower CBZ clearance are reported in homozygous CYP3A5*3/*3 compared to heterozygous CYP3A5*1/*3 [55,56]. On CBZ monotherapy, there was no significant association between patients with CYP3A5*1 or CYP3A5*3 alleles. Conversely, for patients who used CBZ in combination with phenytoin and phenobarbital or VPA, enzyme-inducing antiepileptic drugs, individuals *CYP3A5* wild-type showed a trend of having higher CBZ clearance and lower dose-adjusted CBZ level as compared to individuals carrying the CYP3A5*3 allele [57,58]. Functional *CYP3A5* polymorphisms also play an important role in maintaining steady-state CBZ concentrations; therefore, they can be directly linked to drug toxicity. Indeed, patients homozygous for *CYP3A5* mutant allele (CYP3A5*3/*3) frequently developed CBZ toxicity and had longer half-life and slower clearance rate compared to the wild type [59]. However, there are discordant data in the literature regarding the influence of *CYP3A5* polymorphisms in CBZ metabolism. Several studies reported that *CYP3A5* genotypes did not differ significantly in terms of CBZ dosage requirements, dose-normalized plasma concentrations and clearance. In addition, African Americans carrying CYP3A5*3/*3 had a longer CBZ half-life when compared to CYP3A5*1/*1 or CYP3A5*1/*3 carriers, but the difference was not significant in Caucasians. The clinical impact of the CYP3A5*3 genotypes on CBZ PK should thus be clarified [60,61,62]. 

CBZ and its main active metabolite CBZ 10,11-epoxide are specifically glucuroni-dated by *UGT2B7* enzyme. UGT2B7*2 (802C>T; rs7439366) SNP arises from a C to T transversion at nucleotide 802 of the *UGT2B7* coding region. In a study on 62 epileptic patients in treatment with CBZ as monotherapy, it has been demonstrated that UGT2B7*2 SNP affected steady-state CBZ concentrations. In fact, significative correlation between CBZ levels and drug dose was reported in UGT2B7*2 patients. UGT2B7*1/*2 and *2*/2 patients exhibited lower normalized CBZ concentrations and larger CBZ dose requirements than the wild type subjects. In addition, it has been showed that UGT2B7*3 (211G>T; rs12233719) did not affect the CBZ dose requirement or steady-state concentration. Conversely, other studies did not found any association between *UGT2B7* polymorphisms and drug levels [63,64,65].

Further CBZ bioactivation depends on *CYP2C19*, and formation of 3-hydroxy-CBZ is catalyzed by CYP1A2* 1F (-163C>A, rs762551), both having several functional variants [66]. Carriers of -163C/C and C/A genotypes in *CYP1A2* showed a significant correlation between weight-adjusted CBZ dose and CBZ concentration, as well as carriers with *CYP1A2* -163A/A genotype reported modifications in CBZ PK. In addition to sex and total CBZ daily dose, -163C>A *CYP1A2* polymorphism should be considered as a predictor of CBZ clearance [67,68]. CYP2C19*2 (681G>A, rs4244285) and CYP2C19*3 (636G>A, rs4986893) variants were associated with predisposition to Stevens-Johnson syndrome and toxic epidermal necrolysis (SJS/TEN) after CBZ administration and the absence of CYP3A5*3 (rs776746) might be a protective factor [69,70].

*CYP2C8*, a phase I metabolizing enzyme, is involved in the biotransformation of various drugs, including CBZ. Indeed, *CYP2C8* can promote CBZ conversion into its active metabolite CBZ 10,11-epoxide, even though it is not the main enzyme involved in this pathway. *CYP2C8* enzyme is also inducible and CBZ might induce its expression in a positive feedback. Polymorphisms in *CYP2C8* gene could partially explain inter-individual variabilities observed in response to CBZ treatment. For example, in Caucasians, the CYP2C8*3 (416G>A, rs11572080) variant, the most common nonsynonymous variant, was frequently associated with decreased enzyme activity and increased CBZ serum concentrations. Of great relevance, a CYP2C8*5 (475delA, rs72558196) variant is a rare polymorphism; however, this variant could determine a truncated form of the cytochrome leading to a completely inactive enzyme variant [71,72].

## 4. Genetic Polymorphisms of Drug Metabolizing Enzymes and VPA PK

**Figure 2:**
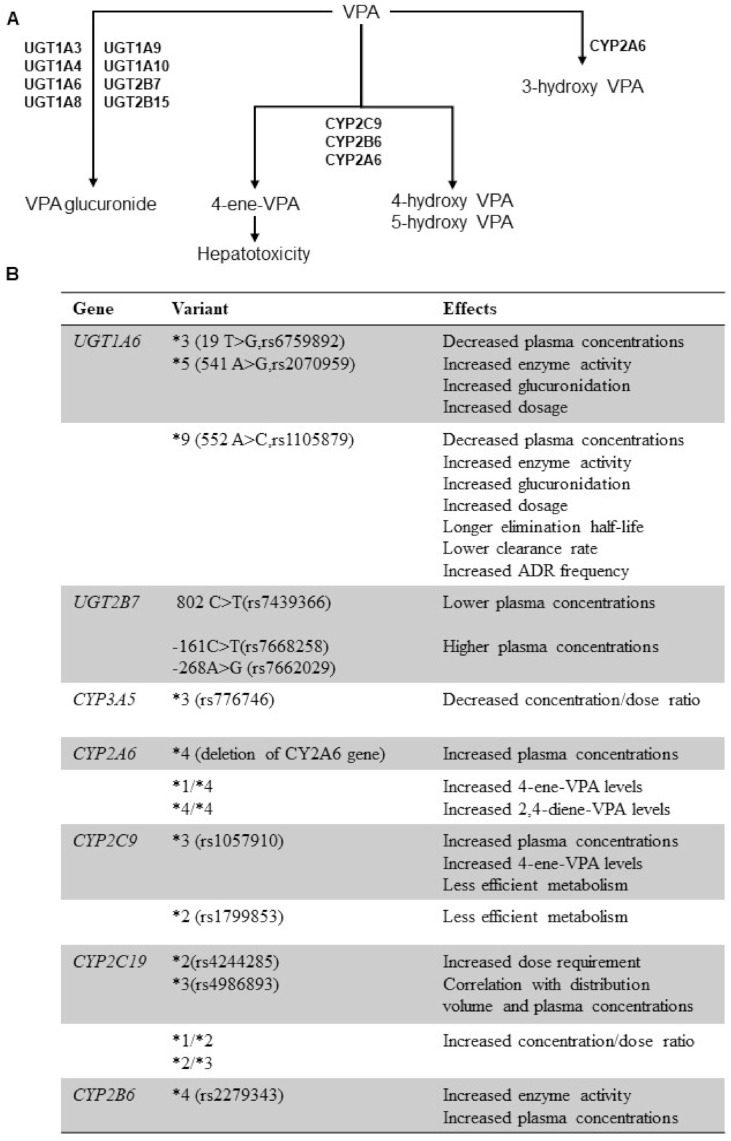
Pharmacokinetics (PK) and pharmacogenomics of valproate (VPA). (**A**) Liver metabolism of VPA by cytochrome P450 (CYP), UDP-glucuronosyltransferase (UGT) enzymes, and relative metabolites. (**B**) Genetic polymorphisms in genes involved in VPA metabolism and their effects on PK of the drug. Abbreviations. (*) allele nomenclature; ADR, adverse drug reactions.

Several factors, such as drug-drug interactions and genetic patient’s background, influence VPA efficacy and tolerability; however, pharmacogenomics studies on VPA are limited and generally include small numbers of patients mainly suffering of epilepsy. Interindividual PK variability can be partly explained by the presence of polymorphisms (mainly SNPs) in genes encoding hepatic enzymes UDP-glucuronosyltransferase (UGT) and CYP (*CYP2C9*, *CYP2A6* and *CYP2B6*) families (Figure 2) [73,74].

Valproate glucuronide is the major VPA metabolite and the reaction of glucuronic acid conjugation is mediated by UGTs, especially *UGT1A* and *UGT2B* including *UGT1A3*, *UGT1A4*, *UGT1A8*, *UGT1A9*, *UGT1A10*, *UGT2B15*, and the most prominent *UGT1A6* and *UGT2B7*. Pharmacogenomic studies have focused on UGT polymorphisms, because genetic *UGT* variants can influence dosage and plasma drug concentrations and can be directly linked to ADR development [75,76].

*UGT1A6* polymorphisms might contribute to inter-individual PK variability; for example, carriers of one of the three common SNPs, UGT1A6*3 (19T>G; rs6759892), UGT1A6*5 (541A>G; rs2070959), or UGT1A6*9 (552A>C; rs1105879), required higher VPA dosages compared to the wild-type, while carriers of a variant in Glutamate Ionotropic Receptor NMDA Type Subunit 2B (*GRIN2B*) -200T>G frequently required lower VPA dosages. These three common *UGT1A6* SNPs displayed a two-fold faster VPA glucuronidation activity compared to UGT1A6*1 genotype. Moreover, patients homozygous for 541A>G and 552A>C *UGT1A6* variants had significantly lower concentration-to-dose ratio during VPA therapy compared to the wild type subjects. Indeed, serum VPA concentrations of A/A carriers was significantly higher than those of A/C or C/C carriers, as also described in Chinese epileptic children [77,78,79]. Carriers of *UGT1A6* 19T>G, 552A>C and 541A>G alleles displayed increased UGT enzyme activity than the wild-type patients, and *UGT1A6* 552A>C carriers showed a longer elimination half-life and a lower clearance rate frequently causing VPA-related ADRs, such as ataxia, liver damage, metabolic changes, tremor, hallucinations, pancreatitis, and weight gain [80,81]. However, the exact influence of those polymorphisms in VPA metabolism and ADR development is difficult to clearly outline as modifications in VPA metabolism were described either when VPA was administered as monotherapy or in combination with CBZ. For example, there were no changes in VPA metabolism in UGT1A3*5 (rs2889861) carriers when exposed to both VPA and CBZ, while lower plasma VPA concentrations was described in carriers treated with VPA as monotherapy. Carriers of UGT1A3*5 allele harboring the A17G mutation had a significant lower VPA plasma concentration. Therefore, UGT1A3*5 carriers required higher VPA dose to reach the therapeutic target within a plasma concentration range of 50–100 μg/ml [82,83,84]. Further studies are needed to identify the real clinical impact of these *UGT1A* haplotypes on VPA dose and concentration-to-dose-ratio, as well as for other UGT polymorphisms, such as *UGT1A4* and *UGT1A9*, although some studies associated *UGT1A9 rs2741049 (I399T>C)* and *rs6731242 (−1887T>G)* SNPs to increasing enzyme activities [85,86,87].

The role of *UGT2B7* in VPA metabolism has also been widely investigated. *UGT2B7* plays an important role in VPA clearance but studies on the clinical impact of *UGT2B7* on VPA metabolism are lacking. In addition, reported data on influence of *UGT2B7* polymorphisms, such as UGT2B7*2 (802C>T; rs7439366), UGT2B7*3 (211G>T; rs12233719) or UGT2B7*4 (1192G>A; rs145725367), on VPA PK are even more conflicting. Carriers of UGT2B7*2 TT and CT genotypes had lower plasma VPA concentrations compared to CC genotype carriers, suggesting that TT and CT carriers might require higher VPA doses to avoid sub-optimal concentrations leading to a lack of drug efficiency. Conversely, *UGT2B7* -161C>T (rs7668258) polymorphism was significantly associated with higher plasma VPA concentrations in epileptic children. Furthermore, *UGT2B7* -268A>G (rs7662029) SNP can affect VPA PK, as AA carriers displayed higher VPA serum concentrations compared to GG carriers, while UGT2B7*3 had a controversial effect on VPA PK [88,89,90,91].

*CYP2C9* is mainly involved in the formation of the hepatotoxic 4-ene-VPA and the inactive 4-hydroxy (OH)-VPA and 5-OH-VPA metabolites. The use of a *CYP2C9*-selective inhibitor, sulfaphenazole, can dramatically reduce the formation of these three molecules; however, sulfaphenazole is more likely a catalyst of 4-ene-VPA formation as opposed to 4-OH-VPA or 5-OH-VPA. *CYP2A6* and *CYP2B6* together account for the formation of 20–25% of 4-ene-VPA, 4-OH-VPA, and 5-OH-VPA. In addition, *CYP2A6* mediates VPA oxidation in 3-OH-VPA, and the use of coumarin, a potent *CYP2A6* inhibitor, significantly reduced 3-OH-VPA formation in human liver microsomes [92,93]. Variants in *CYP2A6*, *CYP2B6*, and *CYP2C9* genes might partially explain the inter-individual variability in VPA PK, as patients with one or two CYP2A6*4 variants (deletion of *CY2A6* gene) had a higher plasma VPA concentrations compared to non-*4 alleles. Moreover, *CYP2B6* genotypes might influence the plasma VPA concentrations: CYP2B6*4 (rs2279343; 785A>G) carriers had higher enzyme activity as opposed to wild type, while protein expression had slightly decreased; or CYP2B6*6 (rs3745274, 516G >T and rs2279343, 785A>G) carriers showed a higher VPA plasma concentration compared with non-*6 alleles. Similarly, patients heterozygous for CYP2C9*3 (rs1057910) and CYP2C9*2 (rs1799853) alleles had increased plasma VPA concentrations compared to CYP2C9*1; however, age and sex might additionally influence drug metabolism together with gene polymorphisms. Indeed, old female patients generally require 30–50 % lower VPA dose compared to the younger males in order to reach therapeutic drug concentrations [94,95]. Allelic CYP2C9*2 and CYP2C9*3 variants were less effective for VPA metabolization, as patients homozygous for CYP2C9*2 or CYP2C9*3 or heterozygous for CYP2C9*2/*3 were identified as slow metabolizer showing reduced efficiency in oxidative VPA biotransformation by liver microsomes. Based on this strong evidence, VPA therapy might be adjusted on *CYP2C9*-status, including polymorphisms and enzyme expression/activity, and it might be particularly recommended in children. Pre-treatment investigation of pediatric patients’ *CYP2C9*-status allowed the optimization of VPA dose, minimizing the exposure to sub-optimal or toxic concentrations thus reducing ADR incidence, such as hyperammonemia [96,97,98,99]. Moreover, CYP2C9*3 allelic variants, CYP2A6*1/*4 and CYP2A6*4/*4 carriers had increased hepatotoxic 4-ene-VPA and/or 2,4-diene-VPA levels compared to the wild type. *CYP2C9* and *CYP2A6* were risk factors for hepatotoxicity by increasing the risk to 7.50 and 5.13 folds, respectively. Therefore, pre-treatment investigation of *CYP2C9* and *CYP2A6* polymorphisms might predict or prevent VPA-related liver dysfunction [100]. VPA metabolism is also influenced by polymorphisms in the Acyl-CoA Synthetase Medium Chain Family Member 2A (*ACSM2A*) gene. Carriers of genetic variants in *ACSM2A* have higher levels of alanine aminotransferase (ALT) and aspartate aminotransferase (AST) compared to wild type [101]. Mutations in a DNA polymerase subunit gamma (*POLG*) gene are also related to increased incidence of serious liver side effects in patients under VPA therapy. Heterozygous genetic variation in POLG was strongly associated with VPA-induced liver toxicity [102]. 

CYP2C19*2 (*G681A*, rs4244285) or CYP2C19*3 (*G636A*, rs4986893) polymorphisms are associated with VPA distribution volume and plasma concentration in epileptic patients [103,104]. Carriers of a CYP2C19*2 variant require higher VPA doses to reach therapeutic target plasma concentrations, indicating that *CYP2C19* is also involved in the VPA pathway. CYP2C19*2 allele carriers required higher VPA doses to reach a plasma concentration of 450 μg/mL, while CYP2C9*13 (rs72558187) variant carriers did not require dose adjustment as there was no correlation between CYP2C9*13 variant and VPA plasma concentrations [105,106]. Mean concentration/dose ratios of VPA were significantly higher in patients with CYP2C19*1/*2 genotype or CYP2C19*2/*3 genotype than in those with CYP2C19*1/*1 genotype. VPA dose for intermediate and poor metabolizers was lower than that employed in extensive metabolizers (*1/*1). The term “intermediate genotype” identified a subject carrying a wild type and a variant allele encoding an enzyme variant with reduced or absent activity (e.g., *1/*2, *1/*3), thus leading to decreased *CYP2C19* activity. Conversely, a poor genotype patient had two alleles with gene sequences encoding for loss-of-function enzymes (e.g., *2/*2, *2/*3, *3/*3) causing a completely absent or markedly reduced *CYP2C19* activity [107]. Japanese females carrying a CYP2C19*2, or CYP2C19*3 variant were more susceptible to VPA-induced weight gain. Moreover, SNPs in leptin receptor (*LEPR*) (rs1137101, 668A/G) and ankyrin repeat kinase domain containing 1 (*ANKK1*) (rs1800497) showed associations with VPA-induced weight gain in Chinese population. Oral clearance of VPA in patients with *LEPR* G668G variant was lower than one observed in patients with *LEPR* -A668A genotype [108,109]. The CYP3A5*3 (rs776746) GG genotypes had decreased concentration-dose ratio compared to AC genotype, suggesting a potential mechanism underlying inter-individual variability in VPA metabolism; however, VPA efficacy was not affected by the presence of the CYP3A5*3 variant [110]. 

## 5. Genetic Polymorphisms of Drug Transporters and CBZ/VPA Response

Drug transporters are membrane proteins involved in the uptake or efflux of drugs. Several adenosine triphosphate (ATP)-dependent proteins, such as the ATP-binding cassette (ABC) transporter superfamily, have been associated with drug resistance, metabolism, and toxicity [111]. Several polymorphisms of the main ABC transporters of P-glycoprotein (*MDR1*, *ABCB1*) and multiple resistance-associated protein 2 (*MRP*, *ABCC2*) influence drug bioavailability and clinical response [112,113]. Alterations in ABC transporter efflux could limit central distribution of many psychotropic drugs, such as CBZ [114]. 

One of the best studied drug transporters is the transmembrane P-glycoprotein (P-gp). P-gp overexpression plays an important role in drug resistance during epilepsy treatment because it is highly expressed in the blood-brain barrier. The polymorphisms in *ABCB1* gene can directly affect the brain uptake as well as the extrusion of antiepileptic drugs, including CBZ. The most studied SNPs in this gene are *C1236T* (rs1128503) in exon 12, *G2677T* (rs2032582) in exon 21, and *C3435T* (rs1045642) in exon 26. The *C3435T* is commonly considered as a critical SNP in antiepileptic resistance. Results pertaining to the assessment of the association of *ABCB1* polymorphisms with the pharmacoresistance are discordant. [115,116]. Patients with drug-resistant epilepsy were more likely to have at *ABCB1* 3435 CC and CT genotypes than TT genotype. The latter was correlated with decreased plasma CBZ levels and lower adjusted drug concentrations compared to 3435 CC carriers [117]. Increased intestinal MDR1 expression was related to low CBZ plasma levels, and genotypes in position 2677 and 3435 of *MDR1* gene might influence CBZ dose requirement; however, allelic association of *ABCB1* *C3435T* was not correlated with increased risk of drug-resistance in large meta-analysis while it was related to refractory epilepsy [118,119,120,121]. Associations between the rs1128503 *ABCB1* synonymous SNP (C>T) and CBZ clearance have been widely described. Patients carrying at least one T allele had a faster drug clearance compared to CC genotype patients. Therefore, pre-treatment pharmacogenetic screening for the presence of rs1128503 polymorphism could be a useful clinical tool to predict responsiveness to CBZ and other antiepileptic agents [122,123]. 

Several studies have identified a correlation between *ABCC2* gene variants and the risk of resistance to antiepileptic drugs with contradictory results. *ABCC2* common variants c.1249G>A (p.V417I, rs2273697) and c.3972C>T (p.I1324I, rs3740066) showed no significant associations with the responsiveness to anticonvulsant drugs, especially CBZ, while only c.-24C>T (5′UTR, rs717620) polymorphism was a risk factor for resistance to therapy in epileptic patients [124]. Indeed, nonsynonymous polymorphism c.1249G>A was associated with reduced CBZ transport but not with drug response in epilepsy patients, while the A-allele of ABCC2 single nucleotide polymorphism c.1249G>A is related to neurological ADRs [125,126,127]. In addition, carriers of *ABCC2* 1249G>A variant were more frequently responders to antiepileptic drugs especially CBZ or oxcarbazepine; conversely, *ABCC2* -24C>T and 3972C>T did not influence CBZ response [128,129]. *ABCC2* rs2273697 polymorphism was significantly associated with 10-hydroxycarbazepine plasma concentrations and might be a predictor of non-responsiveness in non-Asian patients, while *ABCC2* rs717620 CT+TT and rs3740066 778 CT+TT genotypes were over-represented in epileptic patients resistant to CBZ and other antiepileptic drugs in Chinese population, especially female [130,131]. Multidrug resistance-associated protein 1 (MRP1), encoded by the *ABCC1* gene, was overexpressed in neurons and astrocytes of drug resistant epileptic patients, and its inhibition could locally increase drug availability [132]. 

The role of pharmacogenomics for responsiveness to VPA therapy is still controversial as few studies have been carried out and no significant associations have been reported. ABC transporters are considered one of the hottest topics in epileptology. *ABCB1* is one of the most important factors influencing drug transport through the brain-blood barrier. Like *ABCB1*, the *ABCC2* expression was higher in brain-derived endothelial cells from patients with VPA and other drugs resistance [133]. C1236T, G2677T, and *C3435T* polymorphisms in *ABCB1* gene did not contribute to the VPA response in epilepsy; while a CT variant of C129T(rs3213619) polymorphism was related to lower plasma VPA concentrations but not to drug responsiveness [134]. G2677TT and C3435TT alleles, TT, CTT and TTT haplotypes of the *ABCB1* gene were reported to be significantly associated with VPA and CBZ resistance. Patients with CTT and TTT haplotypes of *ABCB1* gene were more likely to show drug resistance compared to patients with CGC haplotype. The presence of C3435TT allele in *ABCB1* gene is a positive predictor of VPA responsiveness in epileptic subjects. Patients with temporal lobe epilepsy displayed a connection between VPA resistance and the presence of CGC haplotype with CC or CT genotypes of rs1045642 and rs1128503 SNPs. An *ABCB1* SNP, rs2032582 (AT and AG), had no clear relationship with VPA and CBZ resistance. ABCB1 1199G>A (rs2229109) polymorphism was frequently found in VPA-related ADRs; in particular, the rs2032582 TA and rs2229109 GA genotypes showed the highest incidence of ADRs and could be identified as the strongest risk factors [134,135,136].

**Figure 3:**
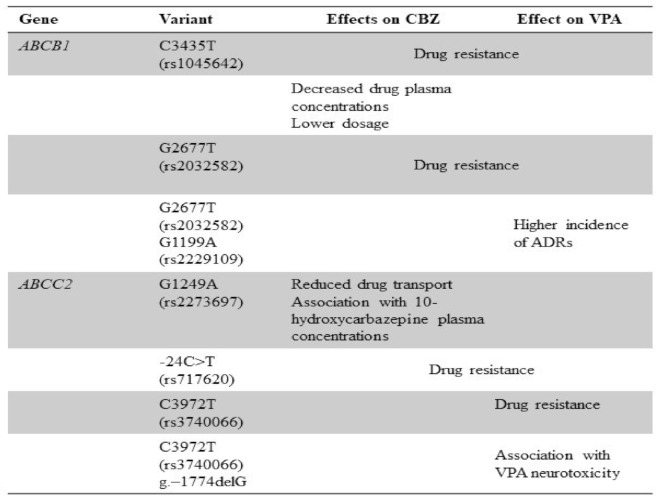
Genetic polymorphisms of drug transporters and carbamazepine (CBZ) and valproate (VPA) response. *ABCB1* (ATP Binding Cassette Subfamily B Member 1), *ABCC2*(ATP Binding Cassette Subfamily C Member 2), ADRs (Adverse Drug Reactions).

The association between *ABCC2* polymorphisms and anti-epileptic drug-resistant epilepsy is discordant. *MRP2* could be overexpressed in resected tissue from epileptic patients suggesting that MRP2 overexpression might be an epiphenomenon in patients carrying *ABCC2* polymorphisms not related to drug exposure. Several studies showed that the *ABCC2* polymorphisms were the risk factors for anti-epileptic drug resistance, more than *ABCB1* variants. In particular, *ABCC2*-24TT or CT+TT genotypes and *ABCC2* 3972 CT+TT genotypes are associated with drug resistance in patients, especially in generalized epilepsy. *ABCC2*−24T allele carriers were at higher risk of anticonvulsant failure probably because of an *ABCB1* up-regulation; however, the exact mechanisms are still unknown. There were no associations between the presence of an *ABCC2* 1249G>A variant and CBZ response [135,136,137,138]. In addition, the presence of G allele and the GG genotype at the g.‒1774delG locus of *ABCC2* resulted in neurological adverse VPA reactions, especially tremor. The haplotype containing GG genotype at this locus was associated to higher neurotoxicity (dizziness, headache, somnolence, diplopia, dysarthria, and tremor) onset in response to the VPA therapy. Other polymorphism associated to VPA neurotoxicity was *ABCC2* rs3740066 in epileptic pediatric population. The strongest risk factor for the *ABCC2* gene was the T allele of rs3740066 [139,140]. The existing findings are summarized in Figure 3. 

## 6. Conclusions

An increasing attention on personalized medicine is oriented to confirm the importance of genetic background on the therapy outcomes. Psychopharmacological treatments keep on having sub-optimal response, for this reason there is a pressing necessity to discover biomarkers of tolerability and efficacy. Pharmacogenomics that investigates the association between specific variants in human genome and variability of neuropsychiatric drugs response have made much progress in recent years. Even though anticonvulsants with mood stabilizer action, such as CBZ and VPA, have been widely used for BD treatment and in various therapeutic areas, it remains difficult to predict the patient’s individual response to therapy in terms of genetic predisposition. Owing to the development of the pharmacogenetic studies, it has been helpful to analyze a large amount of candidate genes affecting the PK variability of VPA and CBZ. 

Most of the studies have focused on the SNPs of CYP and UGT genes, but there have been conflicting results. Genotyping of *CYP2C9*, *CYP2C19* and *UGT1A6* could contribute to the optimization of VPA dosing as affecting its plasma concentrations. Conversely, genotyping of *CYP3A5*, *CYP2C19* and *EPHX1* could stratify the population by higher or lower risk of developing CBZ side effects.

In summary, based on our knowledge, there are potential biomarkers for VPA and CBZ therapy, but they are not so strong to personalize the therapy in patients with BD, and no guidelines have yet been published. However, most trials have been conducted on epileptic patients, especially the pediatric population, and pharmacogenomics information of VPA and CBZ as mood stabilizers were lacking. Further pharmacogenetic research may be needed to better clarify, with the support of Therapeutic Drug Monitoring (TDM), the impact of drug metabolizing polymorphisms in real life settings.
